# Book Reviews

**Published:** 1833-05

**Authors:** 


### CRITICAL ANALYSES.

Quæ laudanda forent, et quæ culpanda, vicissim,

Illa prius, cretâ; mox hæc, carbone, *notamus*.—Persius.

*New Views of the Process of Defecation, and their Application to the Pathology and Treatment of Diseases of the Stomach, Bowels, and other Organs; together with an Analytical Correction of Sir Charles Bell's Views respecting the Nerves of the Face.* By James O'Beirne, m.d., Surgeon Extraordinary to the King, one of the Surgeons of the Richmond Surgical Hospital, Dublin, &c. &c. 8vo. pp. 286. Hodges and Smith, Dublin; Longman and Co., London; and Machlachlan and Stewart, Edinburgh.

The titles of books, like the features of men, are too frequently deceptive, and by no means honest indicators of the interior; but, as far as we know to the contrary, and we have taken some pains to ascertain the fact, Dr. O'Beirne satisfactorily fulfils the expectation held out in his title-page, and presents us with "new views," which are very ingenious in themselves, and *apparently* of more than ordinary value in their practical application. We have been so much gratified by the first perusal of his book, that we sit down with pleasure to go over it a second time, for the purpose of giving our readers an abstract of its contents. This is not often the case with reviewers; for, without the slightest disposition to depreciate the medical literature of the day, it is certain that in most cases the second reading of a work is performed as an act of duty, which is but rarely lightened by the wish of being still better acquainted with its pages. As Dr. O'B. affords us ample materials for occupying all the space we can devote to one subject, we proceed at once to our task of giving a condensed sketch of his views and practice; and we are much mistaken if the knowledge of the work which we shall convey will not lead the profession in general to place it in their libraries, or, at least, to peruse it with the attention it deserves.

Dr. O'Beirne commences by referring to certain "erroneous opinions" which are entertained respecting the filling of the rectum with feces, and the office of the sphincter ani, in the healthy condition of the bowels. Physiologists describe the fecal matter as passing freely from the sigmoid flexure of the colon into the rectum, and gradually distending the latter until, by its pressure, such a sense of uneasiness is communicated to the sphincter ani and muscles of the perineum as to rouse the diaphragm and abdominal muscles to effect its expulsion from the body. It is also believed that the power of retaining the feces, and controlling their discharge, depends exclusively upon the sphincter muscles of the anus. By facts and arguments, Dr. O'B. endeavours, and we think successfully, to shew that these opinions, venerable as they are rendered by time, are quite erroneous. It is admitted that a design to retard the progress of fecal matter, and to convert the large intestines into a depôt for its reception, is obvious throughout the intestinal canal, particularly in the cœcum and colon; and it must be manifest, he says, that if a free communication existed between the sigmoid flexure and the rectum, that design would fail to be accomplished at the point, of all others, at which it was most necessary to have secured its objects; for such an arrangement would necessarily expose the rectum to frequent accumulations, and such as, besides interfering with the ordinary functions of the bladder, would subject the sphincter ani to continued irritation, and thus deprive man of the advantage he enjoys of retaining the alvine contents for hours, or even days, without inconvenience. It is also urged, that the circumstance of nature forming one of her chief depôts for excremental matter in a part of the intestinal canal so close to and continuous with the rectum as the sigmoid flexure is, appears altogether inconsistent with the idea of a free passage between these portions of the canal. We cannot think that the author strengthens his argument by inferring "that the rectum, so far from being open, is firmly contracted and closed," because there is some difficulty felt in administering enemata, and because some force is generally required to discharge the fluid, from the resistance given to its passage upwards.

In answer to this line of argument, we answer, first, that there is generally but little difficulty in giving an enema; and what difficulty there is is sufficiently accounted for by the abrupt manner in which the gut passes over the edge of the sacrum, which juts out into the cavity of the pelvis at its junction with the vertebræ. Neither do we think it is "so exceedingly rare" as he states for the finger of the surgeon to be soiled with feces when he has to examine the state of the rectum. We must observe, too, that in examining bodies after death, we often find the rectum full of feces.

The following argument appears to us quite *ad rem*, and we know not how the facts can be accounted for without admitting the full force of the views Dr. O'B. inculcates.

"The two sphincter muscles of the anus are considerably weakened in the disease called prolapsus ani; in the operation for fistula in ano, these muscles are completely divided, and thereby wholly incapacitated, for a certain time, from acting as sphincters; not only these muscles, but also a portion of the rectum above them, are occasionally destroyed by venereal, cancerous, and other ulcerative processes; yet it rarely happens that the power of retaining the alvine contents is found to be at all impaired in any one of these cases. It is therefore manifest that this could not possibly occur if the passage into the rectum were as free as it is supposed to be, or if the power of retaining the feces, and regulating their discharge, depended solely on the sphincter muscles of the anus." (P. 6.)

We may add too, in the account of the operations performed by

LISFRANC, in which two inches and more of the lower portion of the rectum were removed, no mention was made by our Paris correspondent* of a subsequent involuntary discharge of the feces; and it cannot be supposed he would have neglected to mention so very important a fact. We believe that Mr. MAYO is the only English surgeon who has removed a portion of the rectum by excision, and in his case† the patient, whom we frequently saw at the time in the Middlesex Hospital, was able perfectly to retain solid feces in a short time after the operation, although there was prolapsus of the rectum. Now, in these cases it is very clear that there could be nothing equivalent to a sphincter muscle at the anus. By what means then were the feces retained?‡ Desirous of testing the truth of the inferences drawn from the facts he adduces, Dr. O'B. was led to examine the rectum of a number of healthy persons, at different times in the same day, in order to ascertain its actual state, and, as nearly as possible, the time and manner in which it is filled.

"I proceeded in the following manner, and almost invariably obtained the following results. On passing a stomach-tube to the height of half an inch up the rectum, neither flatus nor feces escaped through it; passing it up about an inch and a half higher, it was still found that nothing escaped, but that it could be moved about freely in a space which, on introducing the finger, was ascertained to be what anatomists call the pouch of the rectum, in a perfectly open and empty state. From the highest part of the pouch to the upper extremity of the bowel, generally a distance of from six or seven to eight inches, it was found that the tube could not be passed upwards without meeting with considerable resistance, and using a degree of force sufficient to mechanically dilate the intestine, which was plainly felt to be contracted so as to leave no cavity for this extent. When the instrument reached, in this way, the uppermost point of the rectum, the resistance to its passage upwards was felt to be sensibly increased, until at length, by using a proportionate degree of pressure, it passed forward rapidly, and as if through a ring, more or less tight, into a space in which its extremity could be moved with great freedom; and as instantly a rush of flatus, of fluid feces, or of both, took place through the tube. In some instances, indeed, it happened that neither gaseous nor liquid matter escaped at this moment, but, in all these, the distinct feel of the extremity of the tube having entered a solid mass in the flexure was communicated to the hand: the instrument, on being withdrawn, exhibited a few inches of its upper extremity covered, and its eyes plugged up, with solid excrement; the person generally went to

* London Med. and Phys. Journal, Nov. 1828, p. 472.—REV.

† Ibid., April, p. 319, and June 1831, p. 510.—REV.

‡ It is scarcely necessary to observe, that we are not here touching upon the question of the propriety or probable advantages of cutting away a portion of the rectum. The result of Mr. Mayo's case was far from favourable, although for some time its progress was very promising.—REV.

stool soon after, and passed a large quantity of solid feces. In every instance where the tube presented the least appearance of feces after being removed, this appearance was confined to that portion of its upper extremity which had entered the sigmoid flexure.

"In this way, I have also examined the rectum of healthy persons in a few minutes after they had passed a stool, and of others at the moment when they felt a moderate inclination to go to stool, and have ascertained that the rectum is in a perfectly empty and contracted state at both of these periods.

"The results of these examinations establish the correctness of the inferences drawn from a number of facts, but in a much more positive and precise manner, for they distinctly prove, first, that in the healthy and natural state, all that part of the rectum above its pouch, is at all times, with the single exception of a few minutes previous to evacuation of the bowels, firmly contracted and perfectly empty, at the same time that the pouch itself, and also the sigmoid flexure of the colon, are always more or less open and pervious; lastly, that the sphincter ani muscles are merely subsidiary agents in retaining the feces." (P. 6.)

At a subsequent part of the work, (p. 45,) the author refers to a fact which might appear to militate against his views. He admits that the rectum may be filled and impacted with hardened feces, and thus cause constipation. Such an occurrence, however, he considers rare, and is easily explained by the circumstances under which it has been observed; for it has never been met with in any but in paralytic, or in very aged, infirm, and sedentary persons. In such subjects, the muscles of the abdomen and perineum, and also the muscular coats of the rectum, participate in the weakly state of the rest of the muscular system; and, when the rectum is filled, neither the same sense of uneasiness, nor the same desire or power to evacuate, is felt, as in stronger or more healthy persons. From these causes, as well as from the listlessness which often leads persons of this description to resist the calls of nature, the bowel, instead of being emptied, and immediately afterwards contracting and closing in the natural way, is left in a more or less distended and open state, so as to no longer oppose sufficient resistance to the descent of the contents of the flexure.

These conditions of the sigmoid flexure and rectum in the healthy state are directly opposite to those in which these intestines are universally considered to be placed, and lead to views very different from those hitherto universally entertained of the physiology of the intestinal canal, and also of the pathology and treatment of intestinal diseases.

In order to remove all rational doubt respecting so important a point, as well as to prepare the way for giving a correct general view of the manner in which the contents are moved through and expelled from the alimentary tube, Dr. O'B. adverts to the structure and disposition of the parts in question, the sources from which they are supplied with nervous influence, and the nature of the functions they have to perform; and he contends they are such, that these parts cannot be in any other than the conditions just mentioned. We cannot extract all the arguments he advances in support of this conclusion; and they are so connected with each other, that an abridgment would be useless to the reader, and injurious to the author. For these details therefore the work itself must be consulted, as well as for the peculiar views entertained by him of the means and process by which the act of defecation is accomplished. Many facts and arguments are advanced, from which it appears very probable that, with the exception of the comparatively-rare instances in which alvine obstruction is the consequence either of the cavity of the rectum being traversed by membranous filaments, impacted with indurated feces, filled by humours and other excrescences, or obliterated by the pressure of tumors, or of displaced or morbidly-enlarged organs external to it, constipation is invariably an effect, and nothing more nor less than an effect of an unusually contracted and impervious condition of the rectum, produced by a more than usually firm and strong action of its own powerful and highly irritable muscular parietes, and maintained by a variety of favourable circumstances, which are explained in the work.

We must not pass over two facts which are of much importance in reference to Dr. O'B.'s views. We give them in his own words.

"While the small intestines can be said to have little more than one muscular coat, and the cæcum and colon but a similar, with the addition of three narrow longitudinal bands, the rectum possesses an internal coat composed of strong fleshy fibres set closely together, and arranged circularly, and an external composed of still stronger and more fleshy fibres arranged longitudinally, also set closely together, and so as to completely surround and cover the internal coat: in addition to these, each of the three longitudinal bands of the sigmoid flexure sends down strong fleshy fibres to be expanded upon and intermixed with those of the proper external coat. It is therefore both an anatomical and a physiological fact, that this intestine exceeds every other part of the intestinal canal in the number and strength of its muscular coats, and consequently in muscular power. Again, this intestine is the only portion of the intestinal canal into which we can trace branches from the regular spinal nerves going directly, and without previously interlacing with filaments of the sympathetic, into its substance; for we can not only trace, but plainly see, that the right and left sacral plexus, which consists of nerves of this description, send the hæmorrhoidal branches in this manner to supply the rectum. It is also, therefore, both an anatomical and a physiological fact, that this is the only part of the intestinal canal which receives nerves directly from the motific and sensific columns of the spinal marrow; and, consequently, that a much higher order of irritability and sensibility is bestowed upon it than upon either the small intestines, the cæcum, or the colon, and that it is thus directly subjected to the influence, both healthy and morbid, of that all important organ." (P. 9.)

Having, by many very ingenious preliminary remarks, established, as he believes, the general caùse of constipation, the author proceeds to consider its curative indications and treatment. In doing so, however, he confines his observations to the disease as it arises from ordinary causes, having before pointed out the manner of managing those cases which form exceptions to the general rule.

"Whenever constipation exists either as an independent affection, which, strictly speaking, can scarcely ever be the case, or as a symptom of other diseases, and whenever it is produced by common causes, it has been proved that the rectum is contracted so as to be rendered impervious, and that, at the same time, the whole of the cæcum and colon, and a portion of the small intestines, are distended with feces and flatus. Now, this being the state of these parts, it is as self-evident as the simplest proposition in nature can possibly be, that the great curative indication is to mechanically dilate the rectum so as to open and form a free communication with the colon, and thereby not only give exit, but circulation, to the matter so confined. This indication at once conducts us to a plan of treatment which will be found to exceed all others in the important points of safety, certainty, and expedition. This plan consists, as the reader must have long since anticipated, in the introduction of a large sized gum elastic tube through the anus into the sigmoid flexure of the colon, and after giving exit to such flatus and fluid feces as may happen to escape, adapting to it a proper syringe, and throwing up such purgative fluids as circumstances may make it necessary to select." (P. 54.)

As much will depend upon proper instruments being selected, we give the description of those employed by Dr. O'B.

"Of late years I have been in the habit of using an apparatus, which consists of two gum elastic tubes and a brass syringe, and may be described thus: one of the tubes is of the largest size, eighteen inches long, open at both ends, bulbous at the upper extremity, and has, at the lower, a brass ferule made to receive and accurately fit the short pipe at the end of the syringe. The other tube is by one-third shorter, and, in all respects, the same as that attached to the horizontal pipe of the stomach-pump. The syringe has, on one side, a spring lever so constructed, that, when firmly pressed upon, it turns a simple stopcock, and forms a communication between the chamber and the short tube at the extremity, while it closes that which had previously existed between the chamber and the short tube situated at one side; in short, it is Weiss's syringe, as improved by the late William Lloyd, an obscure London artist, who added the spring lever, an addition which has perfected, and greatly increased the facility of working the instrument. The manner of preparing and using this apparatus is very simple." (P. 55.)

If, upon the introduction of the tube, the expulsive efforts be violent, which occasionally happens, we are to yield to them, and take advantage of their intermissions to pass it higher and higher. When it has reached the height of eight or nine inches, the opposition to its farther passage will be much increased; but the pressure upwards must be gradually increased until the resistance is completely overcome.* The moment that this occurs, the point of the instrument passes rapidly onwards, and as if through a very narrow ring, and the escape of either flatus or fluid feces, or of both, takes place, gives immediate and greater or less relief to the patient, and assures the operator that the upper extremity of the tube has entered the sigmoid flexure. But, if neither flatus nor fluid feces should escape at this time, the instrument is blocked up with a mass of solid feces. The next step is to throw up the injection. When the tube is withdrawn, a copious and often enormous stool is discharged.

Dr. O'B. assures us that little or no pain is caused by this practice, and that there is no danger if the practitioner is adroit and cautious. He has now employed this treatment for more than nine years, "with the most decided and unexampled success," in almost every variety of disease attended with constipation, and frequently in very desperate cases, and where all other remedial means had completely failed.

"During the last four or five years, also, the practice has been employed by a number of medical men in this city, and in various parts of Ireland; and the reports of cases with which some of these gentlemen have favoured me, mention it in the highest terms of praise. It possesses, therefore, the great and unusual advantage of not coming before the profession in an infant or immature state, or merely on the authority of a single individual, but with all the maturity and weight of ample experience, and sanctioned by the open attestation of a number of eminent and highly respectable practitioners in its favour." (P. 66.)

Dr. O'B. next proceeds to support the doctrines he maintains, by giving detailed accounts of various cases in which the treatment arising from those doctrines proved eminently successful. He has very properly preferred cases of other practitioners to his own, as

* In a note, the author gives an account of the various explanations which have been offered by various surgeons of the cause of this increased resistance at the upper extremity of the rectum. He denies the existence of valvular projections in the mucous membrane, which have been described by Dr. Houston,† and considers the resistance to arise from a spasmodic state of the rectum at this part.

† Dublin Hospital Reports, vol. v. A very brief notice of Dr. Houston's paper is given in our Journal for February 1831, p. 143.—Rev.

often as he could. Many of the cases are really exceedingly interesting. In several, the life of the patient was in the utmost jeopardy, and the rapid, and in some instances almost instantaneous relief that followed the practice recommended, leaves us no room to doubt that the happy result was wholly attributable to it.

The first case mentioned was one of gouty metastasis to the stomach and bowels; the extremities were originally affected. For more than two days there was obstinate constipation. Enemata, given in the ordinary way, failed to produce any good effect. Dr. O'B. felt convinced that the obstinacy of the constipation depended, as he had previously ascertained in tetanus, on an imperviously contracted state of the rectum, and that the introduction of a gum elastic tube alone offered a chance of saving the patient's life. In this opinion Dr. Crampton concurred, and it was introduced in the manner abovementioned. When it had passed to the height of seven or eight inches, "an uninterrupted stream of limpid serous fluid flowed rapidly from the tube, to the amount of three imperial pints." The improvement in the patient was sudden, "and quite magical." She was restored to her usual health in a few days. This case is considered to be unexampled.

"I have altogether failed in discovering an instance of gouty metastasis to the stomach or bowels, in which the mucous membranes of these parts took on, as in this case, the action of serous membranes, and produced an effusion of serum into the cavity of the digestive tube." (P. 72.)

This was one of the earliest cases in which the similarity of the circumstances attending the ordinary mode of administering an enema induced the suspicion, and confirmed the opinion, that the imperviously contracted state of the rectum, which Dr. O'B. had previously discovered to be the cause of the obstinate constipation which attends tetanus, was not peculiar to that disease, but common to the generality of the cases of constipation, and removable by the same means.

The next case mentioned was one of "spinal irritation." Obstinate constipation existed, and could not be conquered by the usual means. When this patient came under Dr. O'B.'s care, she stated that she had taken various purgative draughts, at least one hundred injections had been given, and that, notwithstanding, she had not had a stool of any kind, or passed even flatus per anum, for *nearly six months.* This extraordinary and almost incredible account was confirmed by other persons, who could have no object in practising deception. The treatment already described was had recourse to, and, upon the removal of the tube, "she passed one of the most enormous stools I have ever seen: it nearly filled a large-sized chamberpot, was altogether solid, perfectly natural-looking." For the further progress of this curious case we refer to the work.

Another case of spinal irritation is related principally to illustrate the effects produced by it on the rectum. Dr. O'B. refers those who may be anxious to acquire scientific views and sound practical information upon the subject of irritation of the spine, to the 63d and 64th volumes of this Journal, containing the highly interesting and instructive papers of Dr. W. and Mr. D. Griffin, of Limerick, to which we have had such frequent occasion to refer, and which have obtained their due meed of approbation not only in this country, but throughout the continent, as we perceive by the French, Italian, and German journals.

Dr. E. M'Dowel furnished the author with a case of *mania*, in which there was constipation of the bowels for several days. Purgatives and enemata were given without avail. Dr. O'B.'s plan was tried with immediate and decided relief. Dr. M'Dowel states that he "has frequently employed the tube to remove obstinate constipation, and uniformly with the most decided success."

Mr. Richard Gregory also, the surgeon of the Bellevue Lunatic Asylum, at Finglass, thus gives his opinion.

"As I have found that by far the greater number of the patients who enter the Belle-vue Lunatic Asylum, at Finglass, near this city, have been so obstinately constipated as to resist for a long time the most active and drastic medicines, I have latterly been in the habit of having recourse at once to the use of the tube, and never without immediate success." (P. 90.)

*Narcotic Poisoning.*—A boy was given, by mistake, a strong infusion of stramonium, and Dr. B. attributed his recovery to the repeated and free evacuations from his bowels, which were speedily procured by the use of the tube, &c.

"The stomach and the great nervous centres are all materially influenced by the state of the bowels; all narcotics are possessed of more or less of a constipating quality; and some portion of any poisonous substance taken into the stomach, must necessarily pass into the intestines, in order to see the urgent necessity of repeated evacuations per anum in cases of narcotic poisoning. Besides enabling us to effect this object with more certainty and expedition than by any other means, the introduction of the tube will enable us to inject vinegar and water, an infusion of coffee, or such other fluids as circumstances may render necessary, and so as to make them act upon a much greater extent of the surface of the large intestines, than could possibly be effected by any of the ordinary modes of proceeding. These are all advantages of the highest possible importance to the treatment of cases, in which life or death so often depends upon the promptness and energy with which proper measures are employed.

"These observations appear to be equally applicable to the treatment of other kinds of poisoning; but, as I cannot speak from actual experience of the use of the tube in any of these, I shall refrain from indulging in mere speculation on the subject." (P. 95.)

Two cases of abdominal tumors are related, which afford additional proof of the efficacy of the practice.

*Strangulated Hernia.*—Dr. O'B. states that strangulation is always caused by the prolapsed portion of intestine becoming so distended, generally by the gaseous, and very rarely by the fluid or solid contents, as to be pressed forcibly against the margins of the opening or ring, and to be no longer capable of passing through it, and returning into its natural situation in the cavity of the abdomen. These points have been urged by various surgical authorities.

"But these, and all other writers, have failed to notice or to take advantage of another self-evident and very important fact respecting the condition of an intestine so situated; namely, the fact that strangulation can never be so tight or excessive as to prevent a more or less free passage of air through the hernial tumor. The very circumstance of strangulation being an effect of pressure exerted from within, not to speak of the exceedingly subtle and permeating quality of air, and the force with which it acts, would even show that in such cases the gaseous contents must circulate with considerable freedom." (P. 108.)

It is obvious that the volume of an intestine so situated must be diminished to a certain extent before reduction can be effected; and, when the taxis has failed, the tumor perhaps being tense and painful, and constitutional symptoms urgent, it becomes an important inquiry to consider how far the other means generally employed to reduce the volume of the hernia are capable of answering that purpose; and, if they are not, whether there are any means left untried which would effect it, and rescue the patient from the operation.

The remarks offered by the author respecting the effect of the remedies ordinarily had recourse to for the purpose of reducing strangulated hernia, are ingenious, and not without originality: we shall give them as fully as our limits permit. He admits that ice, and other refrigerating means, are well calculated to lower the temperature of the part, and to condense its rarefied gaseous contents; but, as the intestinal canal is so constructed as to be always nearly filled with air, and as air passes freely in and out of the prolapsed intestine, it is obvious, he argues, that in proportion as the gaseous contents of the tumor become condensed, the space so created will be occupied by flatus coming from the intestines within the abdomen, and that the protruded intestine will thus be left fully as large and as distended as before. "Hence it is that refrigerant applications have so often failed in effecting the objects for which they were employed." Bleeding and tobacco are considered by the author as objectionable means of effecting reduction. In young, weakly, and aged, persons, extensive depletion is not free from danger, and it very often fails. Tobacco is liable to the same objections.

"The question therefore proposes itself, is there any other more certain mode of accomplishing this object, that has not been either proposed or tried, and that is, at the same time, free from all ob-

1

jection? It appears to me that there is. It is this. To introduce a gum elastic tube into the colon, and to leave it there until the large intestines, and eventually the hernial tumor, are emptied of their gaseous contents. If the bowels happened to be well freed, or to contain but a small portion of solid feces, at the time the strangulation took place, success might reasonably be expected from this mode of proceeding; and, if they happened to be loaded with solid matter at that time, it would only be necessary to introduce the tube more frequently, and at intervals of a few minutes between each introduction, first, to empty the sigmoid flexure, secondly, the cæcum, thirdly, the hernial tumor, in order to effect the object in view. It is no argument against the proposed plan, that Mr. Hey,* and other experienced practitioners, have declared that evacuations from the bowels rarely effect the reduction of a strangulated hernia; for it should be remembered that, on such occasions, the rectum contracts the moment it has discharged its contents, and permits but a comparatively small and insufficient quantity of wind to escape, whereas, according to the mode which I propose, the flatus is offered every facility of passing off in a continued stream, and for as long a time as may be found necessary. In one case the contents of the flexure and a portion of the gas contained between it and the cæcum, are evacuated, while the latter is left distended; so that if the bowels should happen to contain much fecal matter, such is the peculiarity of the process by which the cæcum and sigmoid flexure are filled and emptied, and such the rapidity with which the rectum contracts after discharging itself, that repeated stools may take place, without producing any favorable impression on the strangulated portion of intestine. In the other case, no matter how distended it may be, we have it fully in our power to rapidly empty the whole of the intestinal canal, and thus insure the reduction of its strangulated portion." (P. 113.)

As Dr. O'B. has but recently conceived these opinions, he has had but few opportunities of trying their solidity by the test of experience in practice. He adduces some facts, however, which are interesting. For example: Mary Bently laboured under strangulated hernia with the usual symptoms; constipation of four days' standing, stercoraceous vomiting. Purgatives, bleeding, tobacco, and the taxis, failed. Dr. O'B. mentioned his views to the surgeon, who had determined upon operating. The tube was passed into the colon: "the moment this was accomplished, a considerable quantity of flatus escaped through it, and, on quickly laying my fingers flat on the tumor, I found that it was diminished by at least three-fourths of its former size." The surgeon and several pupils perceived this important change in the tumor. In a few minutes all nausea and vomiting had ceased; the pain that remained was inconsiderable. A mild enema was administered by the tube, and feces were discharged. Leeches were applied to the

* Practical Observations on Surgery, by Wm. Hey, F.R.S., p. 117 *et seq.* 2d edit. London, 1810.

abdomen, to remove some remaining tenderness. In a few days a truss was applied, and the patient was discharged free from complaint. We have been obliged to condense the details of this case, but we have retained the leading facts, which to us appear curious and interesting; and, without the least disposition to exaggerate the value of a mode of treatment because it possesses the charm of novelty, we must conclude that the patient was snatched, if not from death, at least from the most imminent danger, by the application of Dr. O'B.'s practice.

Another case of strangulated hernia is given, "in which the rectum was dilated solely for the purpose of removing stricture of that intestine, and was quickly followed by reduction of the hernia." It is the only instance of the kind on record that the author has been able to find; it was published by Mr. Copeland.* A lady had had umbilical hernia for many years. She was seized with violent pain in the abdomen, and vomiting, and had had no motion for seven days. Rupture painful, and incapable of reduction for twenty-four hours. Purgatives had been given to no purpose. By accident she stated that she had been for many years of a very costive habit; that she could never pass her stools without great pain and difficulty, and that they were always very small in size. On examining the rectum, a firm indurated stricture was discovered, about two inches up the intestine, which would not admit the point of a finger to pass it." A rectum bougie of a small size was introduced high up the gut, and retained there about ten minutes. Soon after it was withdrawn there was a copious evacuation of the feces, the vomiting ceased, and the rupture soon returned spontaneously; in short, all her complaints disappeared, and she was in the same state as before the attack." The occasional use of the bougie was persisted in, with permanent relief of the stricture.

Dr. O'B. had frequently read this case without considering its true bearing, or considering its most remarkable feature as more than an accidental occurrence; and neither Mr. Copeland himself, nor any of his reviewers, suspected the connexion between the reduction of the hernia and the dilatation of the strictured rectum, which is perfectly explained by the doctrines and facts adduced by Dr. O'B. The evidence afforded by this case is the more valuable, inasmuch as the relater of it had no preconceived views upon the subject.

Such is the substance of the information the author gives us respecting strangulated hernia. He thus concludes:

"I am not without hopes that he [the reader] will here find both reasoning and facts sufficient to induce him to give the plan a full and fair trial, and that he will not condemn it, should he find it to fail in cases where the neck of the sac is found to form the stricture, where the strangulated intestine is adherent to the sac, or filled and impacted with indurated feces, or where the case is

* On the Rectum, &c. Second Edition. By Thomas Copeland. 1814.

merely one of epiplocele. On the contrary, I am sure that he will give the plan a perfectly fair trial, and that, for this purpose, he will select cases of recently-strangulated enterocele, or entero-epiplocele. In such cases, and they are invariably the most urgent and dangerous, I am sanguine enough to expect that it will often be found to obviate the necessity of performing the operation. It appears also to be peculiarly applicable to the treatment of those rarer kinds of cases in which hernia of an intestine through the diaphragm, or in other situations out of view, is suspected to have taken place, and to have become strangulated." (P. 126.)

Some remarks are made upon the advantages of introducing the tube when unfavourable symptoms exist after the operation, and a case is mentioned in illustration. Some cases of cholic and enteritis are also detailed, which were attended by the author and other practitioners, in which the treatment he recommends was employed with decided and speedy relief.

Mr. O'Hara, resident accoucheur to the Coombe Lying-in Hospital, has furnished Dr. O'B. with cases of puerperal fever, in which it was had recourse to with great advantage; and he states, that "these are but a few out of many cases I have seen illustrative of the efficacy of the tube in those affections to which puerperal women are so subject; and, from the many opportunities I have had during a long residence in a lying-in hospital, the entire charge of which always devolved upon me in the absence of the master, I cannot speak in too high terms of its great value and efficacy."

For Dr. O'B.'s practical views respecting the pathology and treatment of Dysentery, we refer to the work. He promises a work on Tetanus, and assures us that he shall be "enabled to produce a greater number of genuine cases of tetanus, treated successfully, than can be produced by any one author, ancient or modern." The tobacco enema is the remedy in which so much confidence is placed, and Dr. O'B. has published a successful case treated by this means in the third volume of the Dublin Hospital Reports. After so large (we had almost said so injudicious) a promise, we trust, and we are inclined to believe, that Dr. O'B. will not give us reason to put him in mind of the maxim of Horace, "*Quanto rectius hic, qui nil molitur ineptè.*"

Some brief remarks are made upon the subject of Tympanitis and Delirium Tremens.

He concludes that part of his work which puts us in possession of his views of the process of defecation, and their application to the pathology and treatment of diseases of the stomach, bowels, and other organs, by "asserting deliberately that no trace of them is discoverable in any work, ancient or modern; and that the whole subject, so far from being merely improved or remodelled, has been actually created." We are not prepared to gainsay this statement; to us his views and treatment are original, and, in our opinion, deserving of the serious attention of the profession; for, granting even that objections might be urged to some parts of his doctrines, his practice, as stated (we have no reason to doubt) very faithfully in the course of the work, is too strikingly successful not to be put to the test of repeated and careful trials.

We must reserve our notice of Dr. O'B.'s "Corrections of Sir C. BELL's Views respecting the Nerves of the Face," for another opportunity.

---

*Outlines of Human Physiology.* By HERBERT MAYO, F.R.S. F.G.S., Professor of Anatomy in King's College, London; Surgeon to the Middlesex Hospital; formerly Professor of Anatomy and Surgery to the Royal College of Surgeons. *The Third Edition.* 8vo. pp. 478; illustrated with numerous Engravings on copper and wood. Burgess and Hill, London.

WHEN a book has arrived at a *third* edition, it may bid defiance to the reviewer's censure, and cannot need his praise. The public, though occasionally misled, are in general shrewd judges of good books; and, however pleasing may be the plaudits of the journals, there are few intimations that an author can receive of the value of his labours more satisfactory and convincing than the quiet announcement of his publisher, repeated for the second time, that the work is out of print.

We therefore do not, in general, do more than merely notice the appearance of second and third editions; but these Outlines have been so much enlarged and improved, that such a summary dismissal would be scarcely just.

The text, we perceive, even where no great additions of matter have been made, has been in several parts rewritten; and four chapters, viz. those which treat of "the history of the Mental Phenomena and of the Brain; of the Organ of Vision; of the Mechanism of Speech, and of Embryology," assume quite a novel aspect. To these therefore we must chiefly direct the advanced physiologist's attention, assuring him that he will find them to contain much novel and most-curiously-interesting matter, condensed into a small space, and discussed in a masterly manner.

The modern improvements in the art of wood-engraving are so striking, that we are not surprised to find such figures in a great measure superseding the use of copper-plates, and their easy introduction into the text gives them an advantage over every other kind of graphic illustrations. We are glad to find that, in this edition, the figures have been much increased in number: they cannot fail to be of much assistance to the student, although they do not benefit us, from the difficulty of their transference to our pages.

We have named four chapters as having been in great part rewritten, and containing much novel and important matter. Each of these might furnish sufficient for an article; but we must confine our observation to one, and it shall be the last, viz. the account of the modern discoveries in embryology.

It is to BAER that we are indebted for an accurate analysis of the Graaffian vesicles. In 1827 he discovered within these ovula germs, that in the bitch are from $\frac{1}{600}$ to $\frac{1}{250}$ of an inch in diameter, and the structure of the vesicle he states to be as follows:

"1. The peritoneal coat of the ovary.

"2. The proper or cellular coat of the ovary.

"3. The thin outermost coat of the Graaffian vesicle, formed of a close filamentous tissue.

"4. The internal layer, thicker, softer, more opaque, than the outer, from which it is after maceration readily separable. Its inner surface is slightly villous.

"5. The granular membrane, immediately including the humour of the Graaffian vesicle.

"6. The humour itself, slightly viscid, albuminous, pellucid, but inclining to yellow in the most turgid vesicles, containing numerous irregularly-shaped granules, with here and there a globule of oil.

"7. The discus proligerus, or germ disc, composed of closely-coherent granules, which forms the bed in which is placed—

"8. The germ or ovulum, itself a hollow sphere, the crust of which consists of coherent granules.

"There is every reason to believe that Graaffian vesicles are at intervals coming forward during the whole period at which child-bearing is possible; and that they burst in succession, and shed the contained ovula, whether sexual connection takes place or not. It is probable, however, that sexual connection hastens their ripening. A principal difference in the appearance of the ovary of one past the age of thirty, and that of a child, is the scarred and cicatrized surface of the former contrasted with the uniform smoothness of the latter. The cicatrices on the former result, beyond question, from the healing of numerous lacerations of the surface produced by the escape of ovula.

"The appearance termed a corpus luteum is the consequence of the bursting of a Graaffian vesicle. It is formed by a thickening of, or deposit in, the internal coat of the Graaffian vesicle. Baer mentions, that the thickening and the yellow colour sometimes begin to appear before the vesicle bursts.*" (P. 372.)

The history of the development of the embryo is given so succinctly, that it will scarcely admit of any abridgment; we must therefore quote the first part of the section almost entire.

"The detection of the ovulum in mammalia by Baer, which has been mentioned in the preceding section, is a point only in the extensive circle of discovery which has been traced by himself and by other physiologists. According to ancient hypothesis, the development of the embryo was the evolution of parts which pre-existed in the germ. This opinion was first successfully attacked by C. F. Wolff, in a thesis which he published at Berlin in 1759, and in which he not only described a successive production of organs, of the preformation of which no trace existed; but showed that, after parts

* "De Ovi Mammalium et Hominis Genesi. C. E. a Baer. Lipsiæ, 1827."

are first formed, they undergo many important changes in their structure before arriving at their perfect state. The opinions of Wolff, that the parts of the embryo are formed and not evolved, have been verified, and a vast body of knowledge ascertained upon this subject, by the labours of Pander, Meckel, Oken, Blumenbach, Baer, Rathe, Cuvier, Dutrochet, Serres, Rolando, Prevost, Dumas, and of many others, who have joined in creating a new branch of physiological science. I shall endeavour briefly to explain the leading facts which have been thus brought to light. They have an interest greatly beyond what attaches to the narration of phenomena that are simply unexpected and curious. They elucidate to a wonderful degree the affinities of remote families of animals, displaying how for a period their type of formation is the same; and explain the seeming caprice of Nature in instances of monstrous and defective formation, by showing that what appears anomalous is not the substitution of a new type, but the arrest of development, whereby a temporary character is rendered permanent.

"The ovulum bursting from the Graaffian vesicle is received by the fimbriated extremities of the Fallopian tube, and conveyed towards the uterus.

"Baer detected the ovulum in its passage along the Fallopian tube in a bitch. It was of a yellowish-white colour, and consisted distinctly of an outer cortical membrane, or chorion, and of a globule within of a granular structure. Its diameter was the fifteenth part of a line.

"After reaching the uterus, the ovulum grows more rapidly than before. Its structure may be examined with ease, when it is about half a line in diameter. A new element is then visible in it. Upon the surface of the internal globule, a round, opaque, granular, disc is seen, with a dark spot upon its centre. Dr. Allan Thompson states, that he has seen this spot very evident in the ova of the rabbit on the sixth day after impregnation.

"It does not admit of a doubt, that the spot seen on or through the inner membrane of the ovum corresponds exactly with the *cicatricula of the egg*. This conclusion is grounded, less upon their first close resemblance than on the subsequent identity of the parts which replace them, less upon either argument than upon both together. The intermediate state to the two which I refer to, has not been as yet followed out in mammalia. I therefore shall describe the cicatricula in the egg, and its first changes, as typifying what analogy renders certain occurs in all vertebral animals.

"In the unincubated egg of the common fowl, the germ spot, or cicatricula, lies immediately upon the surface of the yolk: it is circular, of a whitish colour, and about one sixth of an inch in diameter. The disc of the cicatricula is formed of different-sized granules united together, and is covered by the proper membrane of the yolk. The central part of the disc is thinner and more transparent than the rest, and has been called the colliquamentum.

"Seven or eight hours after the commencement of incubation, a small dark line may, with the aid of a magnifying glass, be discovered on the upper part of the cicatricula towards the centre of the transparent area. This line or primitive trace, delineated in the adjoined figure, is swollen at one extremity, and is placed in the direction of the transverse axis of the egg. The rounded end is towards the left, when the small end of the egg is turned from us.

"The cicatricula, germ spot, germinal membrane, or blastoderma, resting in immediate contact with the yolk, now expands itself; and at the same time acquiring thickness, it separates, towards the 12th or 14th hour of incubation, into two layers of granules. Of these, the outer, which subsequently gives rise to the osseous, nervous, muscular, and tegumentary systems of the body, is called by Pander the serous layer. The inner, situated next to and in contact with the substance of the yolk, is termed the mucous. After these two layers have appeared, a third is formed in the interval between them: this is called the vascular layer; it is generally in intimate connexion with the mucous layer, and it is to the changes which the two combined undergo, that the intestinal, the respiratory, and the glandular systems appear to owe their origin." (P. 377.)

In and from these three layers of the germinal membrane, all the elaborate structures of the animal body are formed, by a most wonderful series of changes, analogous to those which have of late years been examined with so much attention in plants; the doctrine of vegetable metamorphosis being equivalent to that of animal epigenesis.

It is not our intention to follow out the history of all these changes, but some two or three we cannot pass unnoticed.

Two longitudinal ridges are quickly raised upon the serous layer, and, in about twenty hours, the furrow between these ridges becomes converted into a canal, by the junction of its margins. It is at first open at both ends, but the cephalic extremity, which is the largest, soon becomes closed.

"According to Baer and Serres, some time after the canal begins to close, a semi-fluid matter is deposited in it, which, on its acquiring greater consistence, becomes the rudiment of the spinal chord. The pyriform extremity, or head, is soon after this seen to be partially divided into three vesicles, which, being also filled with a semi-fluid matter, give rise to the rudimentary state of the encephalon. Rolando, Prevost, and Dumas, on the other hand, suppose that the rudimentary state of the brain and spinal chord is constituted by the primitive trace itself, which they affirm exists before incubation has commenced.

"As the formation of the spinal canal proceeds, the parts of the serous layer which surround it, especially towards the head, become thicker and more solid; and before the twenty-fourth hour, we observe on each side of this canal four or five small, round, opaque, bodies. These bodies indicate the first formation of the dorsal vertebræ: in a few hours several more appear, and those first produced become quadrilateral. It is about this time, or from the twentieth to the twenty-fourth hour, that the vascular layer appears.

"Towards the twenty-fifth hour, when the layers of the germinal membrane gradually expanding have covered nearly a third of the yolk, they no longer retain their flat and uniform position, but begin to exhibit various folds, which afterwards serve for the formation of the cavities of the body. That part of the germinal membrane which lies immediately before the cephalic extremity of the embryo is bent down into a fold, so as to make a depression upon the surface of the yolk; and some time afterwards, a similar fold is formed behind the caudal extremity. As these folds of the germinal membrane increase, they gradually turn in below the fœtus at its head and tail; and their margins approach one another under the abdomen, which at this period always lies next to the substance of the yolk. As the layers of the germinal membrane are bent down in a similar manner towards the sides also of the spinal canal, there is formed under each end of the embryo a sac or cavity, which communicates with the yolk by an opening common to both. The two shut sacs thus formed indicate the rudimentary state of the intestinal tube: the anterior corresponds to the œsophageal portion of the intestine, the posterior to the lower part of the large intestine." (P. 380.)

Such are the rudimentary stages of the brain and spinal cord, of the intestines and the other chylopoietic viscera; the containing cavities of which at first are open, and only subsequently enclosed by the folds of the different layers of the germinal membrane; and hence, if any thing arrests the normal development of these parts, monstrosities or malformations result. Such malformations as the exposed bladder, deficient thoracic parietes, cleft palate, hairlip, and others, are thus shewn to be not what they were once considered, *lusus naturæ*, but interruptions of metamorphoses, and arrests of development. Perhaps the sanguineous system affords some of the most curious and striking proofs of this doctrine.

We cannot trace the vascular apparatus throughout all its changes, and must commence the demonstration almost abruptly, at the part which will more especially illustrate our position.

"All the arteries take their origin from one trunk, which springs from the heart. The heart in all vertebral animals is originally one simple elongated sac. The single artery which rises from it branches into five pair of arches, between which the œsophagus lies.

"The single heart and artery, with its branchial arches, are permanent parts in fish; in other vertebral animals they undergo a series of metamorphoses by which they are brought to the characteristic type of the species. The essence of these changes in birds and mammalia is, that the heart spontaneously divides into two separate chambers, the artery into an aorta and pulmonary artery; that some of the arches disappear; others becoming permanent aortic, others permanent pulmonary branches.

"The heart of the dog, at the twenty-first day, is a membranous tube twisted in itself, and slightly divided into an auricle, ventricle, and bulb of the aorta. The wall of the ventricle soon acquires a greater thickness than the rest.

"At a period which is probably between four and six weeks, the rudiments of a septum begin to be perceived in the heart. The septum of the ventricles is produced in two ways: on the one hand it arises as a growth from the base of the ventricles; on the other it originates in the extension and growth of the right ventricle in such a manner as to form a bifurcated extremity to the heart. This appearance begins in the seventh week.

"The external figure which the heart now assumes is permanently retained in several species of mammalia. The heart of the dugong and of the manatee presents in the most characteristic manner this permanent separation of the ventricles. The same is seen in a less degree in the walrus, seal, elephant, and in some of the rodentia.

"The internal construction at present has some resemblance to the permanent character of the ventricles of the alligator. But it is most strangely and strikingly preserved in those human malformations, in which from this period a communication remains throughout life between the two ventricles.

"The nature of the formation of the inter-auricular septum is very obscure, and requires further investigation. The first appearance of an inter-auricular septum is in the direction of the Eustachian valve, which at that time appears to be continuous with the annulus foraminis ovalis; and thus lying before the entrance of the vena cava inferior, causes it to open into the left auricle. This is seen about the fifth week. At the sixth week this septum becomes perforated by many foramina, and by a process of absorption wastes into the Eustachian valve. At the same time a production analogous to the first begins to be formed in the place of the permanent inter-auricular septum. It is remarkable that it is at this period, namely, when the inferior cava begins to be shut out of direct communication with the left auricle, that the pulmonary veins open into it. The valvula foraminis ovalis goes on increasing till the seventh month, when it is already a sufficient flap to cover the orifice of communication.

"At this period of development it is evident that the fœtus becomes fit for pulmonary respiration. The oblique opening, which serves if the embryo remains in utero to transmit the blood from the right auricle into the otherwise empty left, would it is evident become closed, if breathing and a circulation through the lungs were now commenced, and an equal stream of blood were poured into the left auricle as into the right.

"At the time when the aorta gives off its cervical arches, the construction of the adjacent parts participates in the same character, and might more readily be converted into branchial organs than into a higher type. The neck is short and thick, the pharyngeal portion of the intestine is of great size, and its sides are penetrated by clefts.

"Four openings on each side of the œsophagus have been observed in the embryo of the dog, between three and four weeks old; in that of the sheep of three weeks; of the pig at three weeks; of the rabbit on the twelfth day; and in the human embryo of six weeks: in the embryo of the dog, some little time before that mentioned above, only three apertures are found. While three pairs of clefts exist in the sides of the pharynx, there are in the dog, as in the chick, only four pairs of vascular arches; but before the first of these becomes obliterated, a posterior or fifth pair is produced, while at the same time the fourth branchial cleft is formed; so that in the mammiferous animal five pairs of vascular arches and four pairs of clefts exist for some time simultaneously in the sides of the neck.

"A few days after the appearance of the fifth arch, the neck begins to elongate, the apertures are closed gradually on the outside, and the lower jaw becomes more developed; while the vascular arches undergo those changes by which the permanent arterial branches arising from the heart are formed.

"The first and third pair of vascular arches form the carotid and subclavian arteries in mammalia, as in birds, and the second pair seems to be wholly obliterated, or at least gives only a small branch; in mammalia, however, the arch of the aorta, or permanent communicating vessel between the ascending and descending aorta, is formed from the fourth branchial arch on the left side of the œsophagus; so that the order in which the vessels of the head and superior extremities arise is reversed, the right innominata taking its origin before the vessels of the left side.

"The pulmonary vessels appear to be given off by the fourth arch on the right and the fifth on the left side, the fifth on the right being wholly obliterated. While, however, the carotid and branchial arteries become developed from the anterior arches, the pulmonary arches do not continue to carry blood to the root of the aorta, as takes place in those of the bird. The parts by which these arches communicate with the root of the descending aorta (forming in birds the ductus botalli) become gradually obliterated; so that of all the five pairs of vascular arches in the embryo of the mammiferous animal, only one, the fourth of the left side, remains prominent.

"While these changes take place in the pulmonary arches, the bulb of the aorta, from the single cavity of which the pulmonary and systemic vessels arise for some time in common, is divided, so as to form the roots of the aorta proper and pulmonary arteries. According to Meckel, the septum which has separated the left ventricle entirely from the right, appears to be continued onwards into the bulb of the aorta, and thus separates this cavity longitudinally into two compartments. The division of the bulb is, however, imperfect for a time; it advances gradually from the part next the ventricle to that from which the vascular arches rise; so that, while the posterior part is divided, the anterior yet remains single, a communication being left at this part between the aortic and pulmonary roots, which admits of the passage of the blood from the right ventricle into the aorta, when the pulmonary arches are obliterated. When the division of the aortic bulb has just taken place, the arch and descending part of the aorta appear to be a continuation of the pulmonary rather than of the aortic root, the latter appearing to lead only into the vessels of the head and anterior extremities. The ductus arteriosus remains for some time, as at first, short and wide, and has the appearance of being an opening of communication between, or a deficiency in the parietes of the juxtaposed tubes; it afterwards becomes lengthened out and narrowed, and appears during a short period to pass from the aorta to the pulmonary root and aorta continuous with it; but about the tenth week in the human embryo this part is dilated, and forms a more direct communication between the ascending and the descending aorta, and the ductus botalli is now formed by another part, viz. the end of the pulmonary root leading into the arch of the aorta." (P. 414.)

Was ever so satisfactory an account of the malformations of the heart before within our reach? Could the organic causes of the *morbus* ceruleus be physiologically explained, before the discoveries above mentioned were made, by those anatomists who have thus immortalised themselves?

We much regret that our space forbids our making any further extracts. The other parts of this work, which we have left untouched, will be found equally pregnant with interest as those we have slightly scanned. It is no small praise to say, that the additional matter in this new edition is worthy of that to which it has been added, and we expect that we only repeat the general opinion, when we state our belief that MAYO's Physiology which for some years has been, bids fair, by its regeneration, still to continue, a standard work.

---

*A Dictionary of Practical Medicine: comprising General Pathology, the Nature and Treatment of Diseases, Morbid Structures, and the Disorders especially incidental to Climates, to the Sex, and to the different Epochs of Life; with numerous Prescriptions for the Medicines recommended, a Classification of Diseases according to Pathological Principles, a copious Bibliography, with References, and an Appendix of approved Formulæ; the whole forming a Library of Pathology and Practical Medicine, and a Digest of Medical Literature.* By JAMES COPLAND, M.D., Consulting Physician to Queen Charlotte's Lying-in Hospital, &c. *Part I.* 8vo. pp. 336. Longman and Co., London.

IN a previous notice we have already expressed our general opinion of the work; but it at that time reached us at too late a period in the month to allow us to do more than partially examine its contents, and look over one or two of the numerous articles contained in this first part of the most valuable "Dictionary of Practical Medicine" that has hitherto issued from the press. During the interval that has elapsed since we warmly recommended it to the attention of our readers, we have sedulously perused several of the treatises it contains; for it should be understood that this Cyclopædia is not a thing of shreds and patches, one piece gathered here and another collected there, and having no other common bond of union than the thread with which they are sewed into the same cover; but that it consists, as far as it has gone, of a series of well, nay elegantly written essays, containing facts and reasonings collated with much care, and digested by a strong mind.

We are somewhat at a loss which of the monographs to select, as affording, within our limits, such extracts as may justify the encomia we have passed; not that any of the series are barren of examples, but that they are already so far condensed that they almost forbid any further abbreviation. We shall, however, select the essay on the Blood, which occupies sixty-six very closely printed columns. For convenience of reference, this monograph is subdivided into six chief heads, and 161 minor sections. The former relate, I. To the states of the blood in health; II. Exuberance of blood; III. Local determination of blood; IV. Deficiency of blood; V. Morbid effects of loss of blood; VI. Alterations of the blood in disease. The latter subdivisions of course refer to the various subordinate topics included in the six greater sections, and, from their number, it will be at once apparent that the subject has been fully treated.

We shall pass by the three first sections, and make our earliest extracts from the fourth, which we shall quote entire, as it furnishes a convenient illustration of the concise form in which our author writes, and the quantity of information he manages to convey in a few words.

"IV. Deficiency of Blood. Syn. *Anæmia* (from the primitive *α*, and *αἷμα*, blood.) *Bloodlessness.* *Anémie*, Fr. *Der Blutmangel*, Ger. *Dyspepsia Anæmia* (Young.) *Marasmus Anhæmia* (Good.)

"Classif. 3. *Class*, Diseases of the Sanguineous Functions; 4. *Order*, Cachexies (Good.) I. Class, V. Order (Author.)

"34. Defin. *A deficiency of blood in the whole body or in some important organ, not proceeding from natural or artificial hæmorrhage, giving rise to a waxy, bloodless state of the countenance and surface, emaciation, feeble quick pulse, and great languor and debility.*

"35. Defect of blood, bloodlessness, or anæmia, although not of frequent occurrence, is yet occasionally met with, particularly in its less remarkable, or local forms. In connexion with chlorosis it is oftener observed. Cases of anæmia have been recorded by Reiselius, Swhenke, and others; and the disease fully described by Becker, Albert, Janson, Hoffmann, De Haen, Insenflamm, Lieutaud, Hallé, Andral, and several pathologists and practical writers of the present day. I shall first offer a few general observations on local anæmia; and afterwards describe more fully general anæmia and its complications. The deficiency of blood, occasioned by natural or artificial losses of it, is considered under a distinct head.

"36. i. PATHOLOGY OF ANÆMIA. 1st, *Local anæmia.* Deficiency of blood in an organ or part is evidently the result of one or more of the following pre-existing lesions:—*a,* Of diminished influence of that portion of the ganglial or organic class of nerves which supplies the blood-vessels of the organ; *b,* Of defective vital expansion of its capillaries, probably owing to the depressed state of the influence exerted on the vessels by the nerves supplying them; *c,* Of mechanical impediments in the way of a sufficient supply of blood; *d,* Of imperfect developement, or diminished caliber of the arteries by which blood is conveyed to the organ; *e,* Of disease of the organ or part, or an imperfect exercise of its functions; and, *f,* Of unusual flux of blood to other quarters, causing a proportionate diminution of it in others. It is evident that these states are merely local, and are capable of co-existing with other changes affecting the whole mass of the circulating fluid, as respects both its quantity and its quality; and that various disorders of function, according to the particular state on which the anæmia depends, and the extent to which it may exist, will be the consequence.

"37. The organs most subject to this condition of their circulation are, according to M. Andral, the lungs, the brain, the liver, the substance of the heart, the stomach and alimentary canal, and some of the voluntary muscles. To these I would add, the spleen, the ovaria, and the generative organs of the male. In many of these, as in other parts, atrophy is associated with the anæmia; and may be considered, in the majority of cases, as the consequence of it. The symptoms of local anæmia are not always manifested during life; but they frequently are, as I shall have occasion to point out, when considering the morbid conditions of those organs most subject to this change. Thus, in the completest of all the states of local anæmia, as when the obliteration of an artery cuts off all supply of blood to the organ, gangrene will result; frequently, when anæmia is seated on the brain, a form of convulsion is the consequence, with other symptoms stated in the article on this subject (see Brain—*Anæmia of*); and when the ovaria, at the period of puberty, is not supplied with the requisite quantity of blood, owing to deficient influence of the ganglial nerves distributed to the organs of generation, chlorosis, sometimes with more or less of general anæmia, is the constant effect.

"38. 2d. *General anæmia.*—The blood circulating through the body may be most remarkably deficient, in respect both of its quantity, and of the relative proportion of red particles. In many cases in which the absolute quantity of blood in the body is diminished, the globules are still more remarkably deficient, they being insufficient to give the blood its usual deep colour. General anæmia presents itself in practice, 1st, as a primary disease; 2d, as a consequence of pre-existing lesions of some one of those organs which are concerned in conveying the nutritious fluids into the blood, or in the processes of sanguifaction; 3d, associated with other diseases, resulting equally with it from some antecedent affection, the nature of which cannot, perhaps, be readily recognized.

"39. *A.* The primary forms of anæmia, when closely analysed, seem to proceed, 1st, from deficient nourishment; 2d, from deficient vital power, from a torpid or depressed state of the influence of the organic class of nerves on the digestive, assimilating, sanguifying, and circulating organs which they supply.—*a.* The influence of deficient supply of nourishment in producing anæmia may be readily imagined, and instances showing it are numerous. I will merely allude to one: M. Gaspard, whose researches have tended much to advance the state of the pathology of the fluids, has illustrated this part of the subject by observing the remarkable degree of anæmia which existed in a large proportion of the inhabitants of a district devastated by famine, who lived upon grass. A more common and less expected form of general anæmia is that which arises from the injudicious restriction of diet and regimen, during convalescence from acute diseases, particularly those which have required large depletions. Several instances of this state of disease have come before me, and would, I am confident, have terminated in dropsical effusions (§ 44,) or in death, if a different system had not been adopted.

"40. *b.* A torpid state of the organic class of nerves, is one of the most influential, if not the most frequent, antecedent affections to which we can impute this state of the circulating fluid. It is extremely probable that those instances of its occurrence from being shut out from the sun's influence, and the constant respiration of an unwholesome air, arise from the continued privation of salutary stimuli to this important class of nerves, upon which the sanguifying processes depend.

"41. The influence of the sun's rays in promoting all the vital actions, particularly those of organic life, probably from modifying the electro-motive state of the frame, must be evident to all. The good effects of light and air are shown in the vegetable kingdom, the circulating fluids of which cannot be duly formed without exposure to both. The sun's rays diffuse a genial influence through the frames of the aged, and excite the organic and generative functions of the young. It has been observed that those persons who are entirely excluded from the light of the sun, and breathe the close air of mines, are particularly subject to general anæmia. M. Chomel has given a very interesting account of the disease which affected the workmen employed in a coal-mine at Auzain. It commenced with cholicky pains, meteorismus, blackish-green stools, dyspnœa, palpitations, great prostration of strength, followed, in ten or twelve days, by a yellowish or waxy and bloodless appearance of the countenance. The capillary vessels disappeared from the conjunctiva and mucous surface of the mouth; and the pulsation of the arteries could scarcely be felt. The patients complained of palpitations, anxiety, oppression and suffocation on exertion, paroxysms of fever, profuse perspirations, œdema of the countenance, and rapid emaciation. This state continued for six months or a year; and in some cases terminated fatally, with the reappearance of the invading symptoms. Four of these patients were sent to Paris for treatment, and were ordered light nutritious diet, bitter infusions, &c. One of them died; and on dissection, the arteries and veins were found almost void of blood, containing merely a little sanguineous serum; and little or no blood flowed from the parts divided during the inspection. The appearances in this case led Mr. Hallé to prescribe iron-filings in the dose of a drachm daily, with tonics and opium; and, under this treatment, all the symptoms gradually vanished, the capillary vessels reappearing on the surface.

"42. *B.* It is probable that general anæmia will not take place, unless consecutively of remarkable torpor of the vital influence, or of some other morbid condition of one or more of the organs which contribute to the formation of blood. Where the digestive powers and the functions of the liver are weakened, anæmia to a slight degree is not infrequent. Its connexion with chlorosis is merely that of an associated effect of pre-existing depression of the influence of the system of organic nerves. (See *Chlorosis.*) The lungs have been considered by some authors as the organ which is chiefly concerned in the production of anæmia, and consequently have been viewed by them as the seat of hæmatosis, or at least the place where this process is completed. Without disputing that such is the case to a certain extent, I am disposed to view the liver as being equally, if not more, concerned in this function, an opinion long since contended for in my Physiological Notes (see *Appendix to* M. Richerand's *Elements of Physiology*); and consequently as being in many cases very influential in the production of general anæmia. It is probable, however, that other viscera or parts may also give rise to it. Thus it may be admitted that total obstruction of the thoracic duct will occasion it: and I have repeatedly observed it in children affected with various chronic diseases of the viscera of organic life; being here, as in most cases, one of the results of imperfect digestion and sanguifaction, as well as of obstruction to the passage of chyle into the blood. One of the most remarkable cases of general anæmia was recorded by Dr. Combe. In it all the viscera were found nearly bloodless, excepting the spleen; but not diseased in other respects, at least not to the extent of impeding their functions. The thoracic duct and absorbent system were not examined.

"43. The symptoms of anæmia have been nearly all alluded to in the foregoing remarks. I may, however, enumerate them briefly at this place. They consist of a pale, waxy, or blanched appearance of the countenance and integuments, in which the cutaneous veins are scarcely seen; and those which appear are pale, apparently empty, do not fill quickly, or scarcely at all, upon pressure made upon them; and, when emptied, fill very slowly. The conjunctiva has lost its red vessels; the lips, tongue, and inside of the mouth, are pale; the pulse feeble, small, irregular, and readily made still quicker or fluttering upon mental emotion; the patient is languid and very weak; complains of flatulence, borborigmi, and an irregular state of the bowels, with want of appetite, and an occasional nausea; a sense of sinking and syncope, particularly upon assuming the erect posture, followed by palpitations; oppressed, short, hurried, and sometimes gasping respiration; irregular convulsive or spasmodic movements; tremors; œdema of the ancles; and in some cases the more severe symptoms described as following sinking after large depletions (§ 54.) In the more unfavourable cases the patient may be carried off by a fit of syncope upon assuming quickly the erect posture; or by a convulsion; or sink with the symptoms of exhaustion, or with those of effusion on the brain, or in the pleural or pericardial cavities. It most commonly runs into one or more of the complications about to be noticed.

"44. 3d, *Complicated anæmia.* Deficiency of blood, as respects both its diminished quantity and its poor quality, or the defect of red globules, is often associated with visceral disease, of which it is generally the consequence; but it also may give rise to various affections, both functional and organic. That anæmia should be complicated with certain chronic diseases of the liver, mesenteric glands, and absorbent system, chlorosis, &c. may be expected; but that it should give rise to diarrhœa, and to dropsical effusions in various parts, particularly in the shut cavities and cellular tissue, without any alteration of the solids, may not appear so obvious, although admitting of explanation. M. Andral states, that he has observed anæmia in the bodies of persons who had died dropsical; and in persons who had complained of diarrhœa, and profuse perspirations; and very justly considers both the dropsical effusions into the shut cavities and into the cellular tissue, and the exhalation from the digestive mucous surface and skin, as perfectly independent of any local congestion or irritation, and to be analogous to the profuse diarrhœa and perspirations which occur in persons who are brought near to dissolution by long protracted disease. In all such cases, whether attended with effusion into shut cavities or cellular tissue, or with increased exhalation from mucous surfaces, we may consider nearly the same pathological conditions to exist as their principal sources, viz. diminished tone of the exhaling orifices, with lessened vital cohesion of the tissues in which they open; a poor and thin state of the blood, the crasis of which is much lowered; and a more rapid circulation of the remaining fluid.

"45. Anæmia, when existing even in a moderate degree, will often give rise to various functional disorders, which are, however, of no constant character, but differing with the temperament, habit of body, &c. The chief of these are hysterical and epileptic convulsions, palpitations, leipothymia or syncope and palpitations alternately, irregular or anomalous convulsions and spasms, chorea, and various nervous tremors resembling chorea, dyspnœa, sickness or vomiting, œdema of the ancles, diarrhœa, headach, &c., with weak, small, quick pulse; pale, waxy, or doughy state of the countenance; listlessness, flatulent state of the abdomen, gastralgia, cholicky pains, very weak digestion, vermination, and irregularity of the fæcal and urinal evacuations. It will also be followed by atrophy and softening of several of the internal viscera, and general emaciation.

"46. In cases where general anæmia is not excessive, it may be admitted that both inflammation and hæmorrhages may still occur, particularly the latter, from the causes usually producing them; and that they will have a remarkable tendency to terminate unfavorably, owing to the state of the system causing the deficiency of blood, to this defect itself, and to the want of vital resistance, as well as to the incompatibility of most of the means of cure with the state of the constitutional powers and of local action.

"47. *Causes.*—Several of the causes of anæmia have been already alluded to (§ 39—42.) There may be others which have not yet been ascertained. I may state, however, briefly and generally, those which have been usually acknowledged. They consist of insufficient and poor food; excessive secretions and evacuations; masturbation practised early in life, and long continued; long exclusion of the body from the direct influence of solar light and rays; protracted confinement in crowded apartments, in the stagnant and impure air of manufactories, especially when affecting children or very young persons; and the constant respiration of a moist, impure, and miasmal atmosphere, from which the sun's rays are shut out. All these exhaust or depress the vital and nervous powers; whilst some also either cut off the necessary supply to the circulating fluid, or waste its richer constituents. To these causes may be added certain malignant organic diseases, as carcinoma, &c., which, in the latter stages, is always attended with more or less of anæmia; impeded development of organs, particularly those belonging to the generative functions, whose perfect evolution is requisite to the salutary excitement of all the organic actions, especially those of digestion and sanguifaction; and lesions which either impede these latter functions, and interrupt the passage of chyle into the blood, or vitiate these fluids.

"48. *Treatment.*—The most rational and the most successful means that can be employed consist of such as are calculated gently to excite and permanently to promote the organic functions. Of these, the most appropriate appear to be the various preparations of iron, bark, sulphate of quinine, camphor, ammonia, small doses of iodine, æther, &c. combined occasionally with opium, hyosciamus, extract of hops, conium, &c. when the disease is attended with cholicky pains. Conjoined with these, the chalybeate mineral waters, stimulating frictions of the surface, light and digestible food, gentle exercise in the open air, particularly on horseback, and change of air, will be found of much service. During the employment of tonics, due attention should be paid to the state of the secretions and excretions; and, when the bowels are constipated, the more tonic and less irritating aperients be resorted to. Of these, perhaps, the best are rhubarb, and aloes, the aloes-and-myrrh pill, the compound iron pill, &c.

"49. When the state of the system is attended with hysterical, convulsive, and other nervous affections, a combination of tonics and chalybeates, with antispasmodics, as the preparations of valerian, ammonia, zinc, myrrh, extract of hops, galbanum, æther, strychnine, and various others, is indicated. If we have reason to suspect that the anæmia is a consequence of obstruction or of torpor, combined with an enlargement of some organ or part concerned in the formation of blood, the preparations of iodine, the liquor potassæ alone or combined with tonics, the subcarbonate of soda, the boracic acid, and sub-borate of soda, are the best medicines with which I am acquainted.

"Bibliog. and Refer.—*Reiselius*, Miscell. Curios. dec. ii. an. 7. obs. xiv.—*Swhenke*, in *Haller's* Disput. &c., vol. vii.—*Becker*, Diss. Resol. Casûs Pract. Anæmiæ, &c., Leid. 1663.—*Albert*, Diss. de Anæmiâ. Hal. 1732.—*Janson*, de Morbus ex Defectu Liquidi Vitalis. Lugd. Bat. 1748.—*Lieutaud*, Précis de la Médecine Pratique, p. 71. Paris, 1761.—*Hallé*, Journ. de Médecine, &c., par *M. Corvisart*, &c. t. ix. p. 3.—*Gaspard*, in Journ. de Physiol. Experiment. &c., t. i. Octobre 1821.—*Chomel*, art. *Anémie*, Dict. de Méd. t. ii. p. 238.—*Roche*, art. *Enémie*, Dict. de Méd. et Chirurg. Pract., t. ii. p. 372.—*Andral*, Clinique Médicale, t. iii. p. 558; also his Pathological Anatomy, translated by *Townsend* and *West*, vol. i. p. 91.—*Combe*, Trans. of the Med. and Chirurg. Soc. of Edin., vol. i. p. 194." (P. 172.)

In the section which treats of the "Morbid Effects of the Loss of Blood," we find much sound reasoning and good practical advice. We can, of course, make but two or three short extracts.

"62. ii. Of excessive Loss of Blood in the course of various diseases.—There are two important considerations which should not be overlooked in practice; viz. that in many diseases, apparently attended with excitement, we shall meet with cases in which the actual quantity of blood in the body is much less than usual; and in various others, bloodletting will often not be borne, although seemingly indicated, and although the quantity of blood in the frame be not lessened. In illustration of the former of these, I may state that many years ago I had an opportunity of remarking minutely the appearance on dissection of a man of middle age, and somewhat fat, who had complained of an acute and painful disease, obviously functional, for which he had been blooded only twice on successive days, and on neither occasion to above thirty ounces; and yet the symptoms of excessive loss of blood appeared, from which he died in twenty-four hours after the second depletion. The most careful examination could detect no organic change, excepting the remarkably bloodless and pale state of all the viscera. Even the brain was less vascular than usual. That in various diseases, unattended by diminution of the circulating fluid, depletion will produce marked symptoms of depression and sinking, owing to the state of the vital power being insufficient to accommodate the vessels, by their tonic or vital contraction, to the reduced bulk of the blood, is well known, and has been fully discussed in the articles on *Adynamic Fevers*, *Erysipelas*, and *Puerperal Fevers;* in which, as well as in puerperal mania, and various other acute diseases, large vascular depletion is often most injurious.

"63. *A. Of excessive loss of blood in diseases of excitement.*— The morbid effects of large depletions will necessarily vary with the nature of the disease in which they are employed. When carried too far, in cases of excitement, where the nervous or vital power is not depressed, and the blood itself rich or healthy, reaction generally follows each large depletion, and thus often exacerbates or brings back the disease for which it was employed, and which had been relieved by the primary effects of the evacuation. This is more remarkably the case in acute inflammations of internal viscera, particularly of the brain or its membranes. Thus, every observing practitioner must often have noticed that a large depletion, when carried to deliquium, will have entirely removed the symptoms of acute inflammation when the patient has recovered consciousness; and that he expresses the utmost relief. But it generally happens that the inordinate depression, the very full syncope which is thought essential to the securing of advantage from the depletion, is followed by an equally excessive degree of vascular reaction, with which all the symptoms of inflammation return; and the general reaction is ascribed entirely, but erroneously, to the return of the inflammation, instead of the latter being imputed to the former, which has rekindled or exasperated it, when beginning to subside. The consequence is, that another very large depletion is again prescribed for its removal; and the patient, recollecting the relief it temporarily afforded him, readily consents. Blood is taken to full syncope, again relief is felt, again reaction returns, and again the local symptoms are reproduced; and thus, large depletion, full syncope, reaction, and the supervention on the original malady of some or all of the phenomena described above as the consequence of excessive loss of blood, are brought before the practitioner, and he is astonished at the obstinacy, course, and termination of the disease, which, under such circumstances, generally ends in dropsical effusion in the cavity in which the affected organ is lodged; or in convulsions, or in delirium running into coma; or in death, either from exhaustion, or from one of the foregoing states; or, more fortunately, in partial subsidence of the original malady, and protracted convalescence. Such are the consequences which but too often result, which I have seen on numerous occasions to result, when bloodletting has been looked upon as the only or chief means of cure, the 'sheet-anchor' of treatment, as it too frequently has been called or considered during the last twenty years.

"64. *B. Of the mode in which excessive loss of blood in disease may be best avoided. Method of conducting bloodletting.*—From the above it will appear obvious that, if bloodletting were better managed, and directed so as to make an impression on the local ailment, but in such a manner as to avoid being so readily followed by the reaction which reproduces the malady for which it was employed, great advantage in practice would result, and much less blood require to be removed, even in the most acute cases. *a.* In order to accomplish this, I have long been in the habit, and have inculcated it in my lectures on the practice of medicine, from 1824, of directing the following mode of practice, when large bloodlettings were required in the treatment of visceral inflammation: The patient should be either in bed or on a sofa, and in the sitting or semi-recumbent posture, supported by several pillows. The blood is to be abstracted in a good-sized stream, and the quantity should have some relation to the intensity and seat of the disease, and the habit of body and age of the patient, but chiefly to its effects; it should flow until a marked impression is made upon the pulse, and the countenance begins to change. Further depletion must not now be allowed; but the finger should be placed on the orifice of the vein, the pillows removed from behind the patient, the recumbent posture assumed, and the arm secured. Thus a large quantity of blood may be abstracted, when it is required, without producing full syncope, which should always be avoided; and when a large mass of this fluid is either unnecessary, or might be hurtful, the speedy effect produced upon the pulse and countenance by the abstraction of a small quantity will indicate the impropriety of carrying the practice further. In this manner I have often removed about forty ounces of blood, where large depletion was urgently required, before any effect was produced upon the pulse, but always carefully guarding against syncope; and, by the subsequent means used to prevent reaction, no further depletion has been required." (P. 177.)

"68. Under no circumstances ought a patient to be blooded whilst his head is nearly on the same level with the trunk; and the utmost care should be taken in having recourse to venesection in cases of dilatation of the cavities of the heart, particularly those of a passive nature. It is seldom necessary in such cases; and if circumstances should arise to require it, the blood should be taken, in the manner I have calculated (§ 64), from a small orifice and to a small extent. In the majority of cases, the state of the venous circulation, if duly examined, furnishes some information as to the quantity of blood in the system, and therefore sometimes becomes a valuable guide to bloodletting in some doubtful cases." (P. 178.)

The sixth and last section is the most copious of the whole, and it contains an admirable digest of what has been discovered both by ancient and modern physiologists, with regard to that very obscure problem, "the alterations of the blood in disease." It, however, will not admit of abridgment; and we again recommend it, and the other articles with which it is accompanied, to the attentive perusal both of students and practitioners.

